# HIV-1 Tat: Role in Bystander Toxicity

**DOI:** 10.3389/fcimb.2020.00061

**Published:** 2020-02-25

**Authors:** David Ajasin, Eliseo A. Eugenin

**Affiliations:** Department of Neuroscience, Cell Biology, and Anatomy, University of Texas Medical Branch, Galveston, TX, United States

**Keywords:** connexin, HIV-1, Tat, HIV-1 latency, cardiomyopathy, neuro-HIV

## Abstract

HIV Tat protein is a critical protein that plays multiple roles in HIV pathogenesis. While its role as the transactivator of HIV transcription is well-established, other non-viral replication-associated functions have been described in several HIV-comorbidities even in the current antiretroviral therapy (ART) era. HIV Tat protein is produced and released into the extracellular space from cells with active HIV replication or from latently HIV-infected cells into neighboring uninfected cells even in the absence of active HIV replication and viral production due to effective ART. Neighboring uninfected and HIV-infected cells can take up the released Tat resulting in the upregulation of inflammatory genes and activation of pathways that leads to cytotoxicity observed in several comorbidities such as HIV associated neurocognitive disorder (HAND), HIV associated cardiovascular impairment, and accelerated aging. Thus, understanding how Tat modulates host and viral response is important in designing novel therapeutic approaches to target the chronic inflammatory effects of soluble viral proteins in HIV infection.

## Introduction

In 2017, it was estimated that there are about 38 million people living with Human Immunodeficiency Virus-1 (HIV) worldwide, with about 1.8 million new infections in the same year (UNAIDS). Despite these large numbers, HIV-infection, and associated deaths are decreasing mainly due to the advent of combination antiretrovirals (ARVs). The longer lifespan of HIV-infected individuals on antiretroviral therapy (ART) opened new challenges resulting in chronic diseases that are represented by accelerated aging diseases such as dementia, Alzheimer's/Parkinson like diseases, stroke, and cardiovascular diseases.

HIV is the causative agent of acquired immunodeficiency syndrome (AIDS). Currently, AIDS is only observed in countries with minimal infrastructure for detection, follow-ups, and ART access. AIDS corresponds to a significant decline in CD4^+^ T cells levels below the critical threshold of 200 cells/mm^3^, resulting from immune exhaustion and inability of the immune system to replenish CD4^+^ T cells faster than the rate of HIV-mediated depletion of the same cells, thereby resulting in opportunistic infections and cancer-related diseases (Lackner et al., 2012). While AIDS is not common in most developed countries due to access to quality health care the same cannot be said for some under-developed countries where access to quality healthcare and therapy is limited.

The development and introduction of combinatory antiretroviral therapy (cART), which is a combination of three to four antiviral drugs targeting multiple stages of the viral replication cycle is critical in inhibiting HIV replication, maintaining CD4^+^ T cells at near-normal levels, and preventing onset of AIDS which has resulted in longer live-spans of the HIV-infected population (Moutouh et al., 1996). Despite these successes, cART does not cure HIV because the virus, early in the acute phase of the infection, colonizes different tissues, infecting resident cells that have a longer half-life than circulating immune cells and remain for extended periods in a latent state by becoming silent and preventing immune detection. These silent nature of the circulating and tissue associated viral reservoirs become evident because upon ART interruption, the virus rebounds or re-emerges from tissues. Thus, the new focus of the HIV field is on identifying reagents and strategies to cure HIV-infection by eradicating the viral reservoirs (Deeks et al., 2012; Barre-Sinoussi, 2013; Barre-Sinoussi et al., 2013; Abulwerdi and Le Grice, 2017; Cafaro et al., 2018). In addition, the success of cART and maybe the presence of viral reservoirs has exposed several HIV-associated chronic pathological conditions that were not very apparent before the development of the therapy. Currently, people with HIV live longer with nearly undetectable viral loads (Barouch and Deeks, 2014), however, conditions such neurocognitive and cardiovascular impairments persists in a considerable proportion of the population (Fiala et al., 2004; Coll et al., 2006; Becker et al., 2009; Clifford and Ances, 2013; Anand et al., 2018; Alcaide et al., 2019; Estrada et al., 2019; Eyawo et al., 2019). It is estimated that about 50–60% of people with HIV develop HAND and the incidence of cardiovascular disease is probably higher in the same population. Hence, there is a need for more research to understand these HIV-associated impairments and how to eradicate them. We propose that some of these toxic and chronic effects are mediated by soluble viral proteins, including Tat, that continue to be produced and circulated despite adherence to cART.

## HIV-1 Replication Cycle

HIV is a lentivirus that infects CD4^+^ cells that expresses both or either of the coreceptors (CCR5 or CXCR4). Infection starts with the virus binding to CD4 receptors on the surface of susceptible cells via the viral gp120 protein (Dalgleish et al., 1984; Maddon et al., 1986). The interaction between the virus gp120 and host protein CD4 induces conformational changes that expose the V3 loop in gp120. The V3 loop binds either CCR5 or CXCR4 and causes the exposure of gp41, particularly, the fusion peptide. This exposed fusion peptide inserts into the plasma membrane of the target cell leading to the tethering of the viral and target membranes. gp41 folding drives the two membranes closer to each other, then the membranes mix and form fusion pore through which the virus core is delivered into the cytoplasm. The delivered viral core undergoes reverse transcription into pro-viral DNA, and translocation to the nucleus occurs (the sequence of this is still debated). The translocated pro-viral DNA is incorporated into the chromosomes, and it is transcribed by the host RNA polymerase II into viral mRNA, which can be un-spliced, singly spliced, or multiply splice. The multiply spliced mRNA is translated into of the accessory and regulatory proteins, the singly spliced is translated into the envelope (env), or gp160 (gp120 and gp41), and the un-spliced mRNA is either translated into Gag or serves as the genomic RNA (Schwartz et al., 1990). All viral components are assembled at the plasma membrane into immature virions. These virions bud from the plasma membrane undergo maturation that allows for the cycle to be repeated.

## HIV-1 Genome Structure and Genes

HIV genome is ~10 kb long, and it is flanked by long terminal repeats (LTRs) on both the 5′ and 3′ ends. The 5′ LTR serves as the promoter region for transcription of viral genes, and it encodes sequences for multiple transcription factors like NFκB, Sp1, NFAT, and others. Also, it encodes the Tat-binding trans-activation response (TAR) elements, which is critical for the transcription of the integrated HIV proviral DNA. Other regions of the genome encode for: Gag polyprotein which is the structural protein of HIV made up of Matrix (MA), Capsid (CA), Nucleocapsid (NC), and p6; Pol polyprotein that codes for the enzymes: Reverse Transcriptase (RT), Integrase (IN), and Protease (Pr); Envelope (Env) for gp120 and gp41; and other accessory and regulatory proteins. Transcription of these HIV mRNAs to make the different viral proteins starts with Tat activity. Hence, Tat is a critical protein for HIV.

## HIV Tat Protein

**Figure 1:**
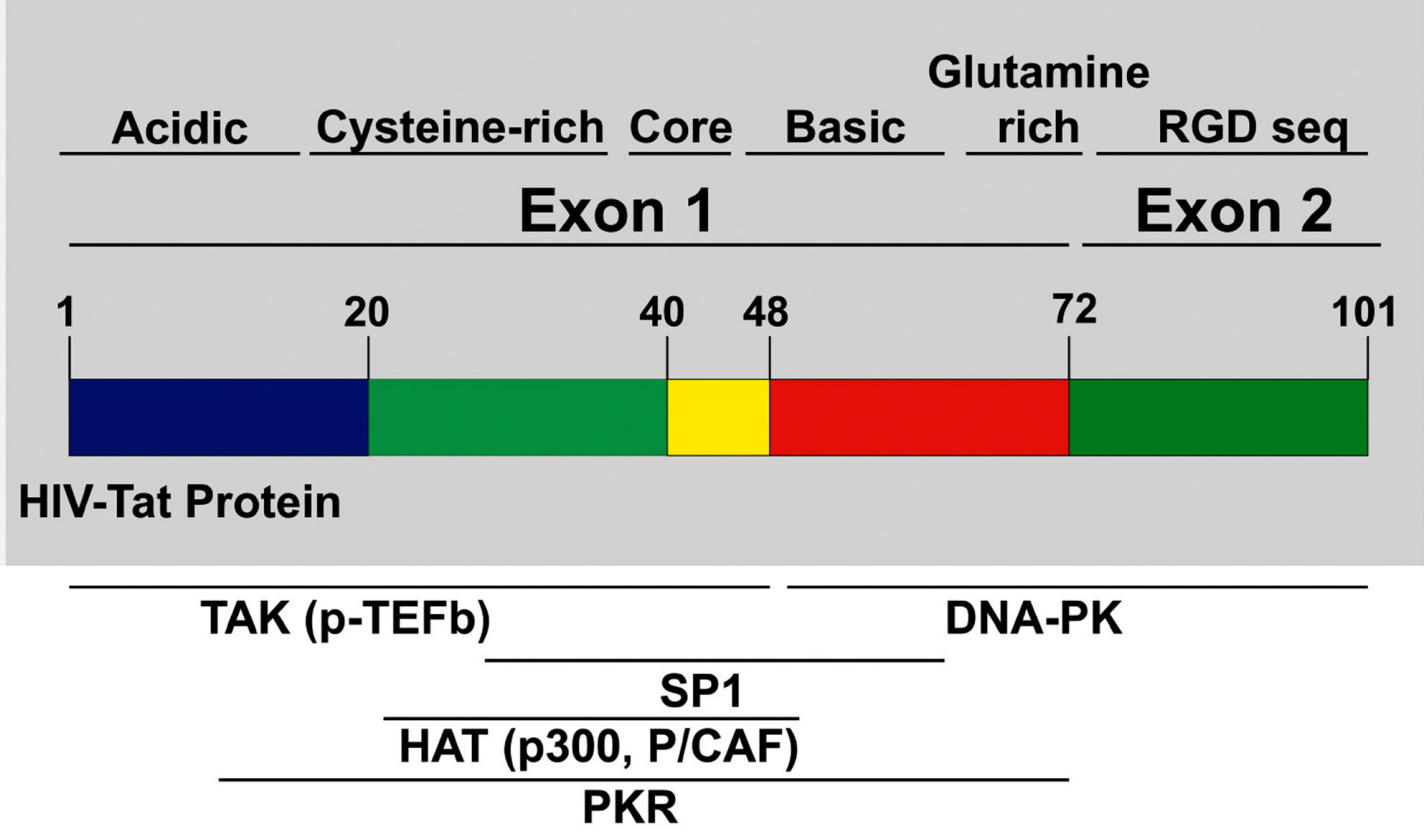
HIV Tat domain structures: tat protein is encoded from exons. Exon 1 encodes for multiple domains like the cysteine-rich, core, the basic domain, and the glutamine-rich domain. Exon 2 encodes for the Arginine rich domain. Most of the p-TEFb complex interactions, as well as other interactions necessary for transcription of HIV provirus, are mediated by domains in Exon 1.

Tat protein is a small protein, 86–102 amino acids (aa), and it is predicted to have about 14–16 kDa molecular weight (Dayton et al., 1986; Bohan et al., 1992; Debaisieux et al., 2012; Clark et al., 2017). Tat is encoded by two exons spliced together, as shown in Figure 1. The first exon (1–72 aa) is conserved within most of the HIV subtypes and well-characterized but the second exon (73–102 aa) is not as well-conserved as the first exon, and it contains an RGD binding site (Chiozzini and Toschi, 2016).

Multiple studies (Garcia et al., 1988; Ruben et al., 1989; Albini et al., 1998a; Benelli et al., 1998; Monini et al., 2012) have shown that Tat protein contains multiple domains. The N-terminal domain (1–48 aa) is critical for activating transcription of HIV genomic DNA due to the cysteine-rich motif required for dimerization, protein structure stabilization, metal binding, and the hydrophobic core motif that is known to be critical for its transactivation activity through its binding to transactivation response RNA element (TAR) of the newly transcribed HIV genomic RNA (Chiozzini and Toschi, 2016). The arginine-rich or basic domain is the second domain of Tat (49–58 aa) that is important for localizing Tat to the nucleus, binding of Tat to TAR element, and the internalization of Tat protein into bystander cells by its interaction with surface proteins such as heparan sulfate proteoglycans (Tyagi et al., 2001; Ruiz et al., 2019). The next domain is the glutamine-rich domain, which has been linked to Tat interaction with the TAR element and important for the Tat-apoptosis function (King et al., 2006; Loret, 2015). The second exon of Tat is mostly important for the replication of HIV in both T cells and macrophages. The first motif in the second exon is the arginine-glycine-aspartic acid (RGD) sequence, which is important for Tat interaction with integrins.

Tat is known to be the viral protein that controls HIV-transcription (Dayton et al., 1986). First, transcription starts with short and abortive transcription by RNA Polymerase II (RNA POL II) (Kao et al., 1987). The short transcripts are translocated to the cytoplasm where they are translated into Tat and Rev proteins. Newly translated Tat enters the nucleus to activate RNA POL II driven transcription elongation by binding to P-TEFb (complex made up of CDK-9, Cyclin T1) (Karn and Stoltzfus, 2012; Asamitsu and Okamoto, 2017; Asamitsu et al., 2018). The Tat-P-TEFb complex binds the TAR element on the RNA, which leads to increased processivity of RNA-POL II. Apart from this interaction with P-TEFb, Tat also recruits histone acetyltransferases (HATs) like CNP/p300 complex to the viral promoter to activate the acetylation of nucleosomes to promote transcription of HIV RNA (Nekhai and Jeang, 2006; Vardabasso et al., 2008).

When there is no activation of the above complex, the infected cell becomes latent, as seen in resting CD4^+^ T lymphocytes and monocytes-macrophages, which are called viral reservoirs (Karn, 2011; Donahue et al., 2012; Kumar and Herbein, 2014; Kamori and Ueno, 2017; Khoury et al., 2018). The establishment of latency or generation of viral reservoirs is mainly due to several cellular processes that restrict access of the transcription machinery to the HIV-1 promoter region (Karn, 2011). Latency also can be established by proviruses that carry transactivation-defective tat sequence (Tat-C22G) that has been shown to prevents effective expression of viral proteins (Wang et al., 1996), which eventually represses Tat expression and/or function. Recent findings have demonstrated that latently HIV-infected cells can be reactivated by adding Tat and one of the proposed cure strategies against preventing reactivation of latently infected cells is to repress the provirus and prevent access of Tat to the promoter region of the provirus by using Tat inhibitors (Easley et al., 2010; Karn, 2011; Mbonye and Karn, 2011, 2017; Siliciano and Greene, 2011; Deeks et al., 2012; Donahue et al., 2012; Desplats et al., 2013; Tabarrini et al., 2016; Kamori and Ueno, 2017; Asamitsu et al., 2018; Khoury et al., 2018). However, our focus for this review will be on how Tat can be released from infected cells and taken up by both uninfected and HIV-infected cells mimicking the transcriptional and cytotoxic effects of the protein. It is important to note that none of the current cART prevents the transcription and/or synthesis of Tat protein.

Tat is secreted from HIV-infected cells and perturbs both HIV-infected and uninfected cells in the surrounding microenvironment (Chang et al., 1997; Debaisieux et al., 2012; Bagashev and Sawaya, 2013; Berks et al., 2014; Clark et al., 2017). Most of the scientific communications described two different actions of this early viral protein, a nuclear and a membrane/cytoplasm associated function. Secretion of Tat into the extracellular space leads to the generation of B cells and T cells antibody responses, as seen in about 20% of HIV-infected patients that are seropositive for Tat antibodies, and studies have shown that seroconversion is associated with low to no progression to AIDS, thereby, indicating the important role Tat plays in HIV-1 replication, infection, and pathogenesis (Re et al., 1995; van Baalen et al., 1997; Allen et al., 2000; Rezza et al., 2005). It also shows that getting rid of extracellular Tat, as is the case with these generated antibodies, might help to slow down the progression of HIV disease. Most of the antibodies against Tat maps to the epitopes in the basic and cysteine-rich regions in the N-terminus (Re et al., 1995; Rezza et al., 2005; Chiozzini et al., 2014).

Tat uptake has been shown to lead to the activation of several transcription factors through phosphorylation or other means (Montano et al., 1997; Karn and Stoltzfus, 2012). Tat-induced activated transcription factors like Sp1, NF-κB, and others have been shown to modulate the expression of both HIV and host genes. Several cellular genes, mostly pro-inflammatory cytokines (like TNF-α, CCL2, IL-2, IL-6, and IL-8), adhesion molecules and sometimes, pro- and anti-apoptotic factors (Albini et al., 1998b; Eugenin et al., 2005; El-Hage et al., 2006a; Lawrence et al., 2006; Youn et al., 2015) is upregulated by these transcription factors via Tat activities. Soluble Tat, in the absence of the virus, has been shown to cause: induction of apoptosis, the release of neurotransmitters, oxidative stress, and inflammation. These mechanisms will be discussed below. Tat modulation of several genes involved in the processes, as mentioned earlier, all contribute to chronic inflammation in people with HIV, and it has been linked to several comorbidities observed in the HIV-infected population, including HIV-associated neurocognitive (HAND) and cardiovascular impairment (Clifford and Ances, 2013; Anand et al., 2018).

Secreted Tat protein has been detected in cerebrospinal fluid (CSF), sera and tissues of HIV-infected people, even in individuals with no detectable viral load (Ensoli et al., 1990; Westendorp et al., 1995; Xiao et al., 2000; Choi et al., 2012). Since most of the HIV-infected individuals are on ART with minimal to undetectable viral replication, still, circulating levels of Tat, in some cases can reach nanomolar concentrations (Ensoli et al., 1990; Westendorp et al., 1995), probably due to the ongoing abortive viral transcription that favor transcription of early genes like Tat (Bachani et al., 2013).

## Tat Release From HIV-Infected or Transfected Cells

Tat accumulates at the plasma membrane of both HIV-infected or Tat-transfected cells, and due to the cytotoxicity of Tat protein, infected, or transfected cells release most of the cellular Tat as demonstrated in infected or transfected CD4^+^ T cells (Chen et al., 2002; Rayne et al., 2010). Tat is secreted by an alternate mechanism that is dependent on its ability to bind to phosphatidylinositol 4,5-bisphosphate (PI (4,5) P2) through a tryptophan residue (W11) and negatively charged patches on the inner leaflet of the plasma membrane (Chang et al., 1997). In addition to secretion, Tat is biologically active both extracellularly and intracellularly when taken up, as seen in multiple studies that performed trans-well transactivation of reporter genes under the control of HIV-1 LTR (Fittipaldi et al., 2003; Vendeville et al., 2004; Fittipaldi and Giacca, 2005; Ruiz et al., 2019).

## Tat Uptake by Bystander Cells

The secretion of Tat into the extracellular environment can lead to viral and cellular responses. As mentioned earlier, several groups showed that Tat could act as a viral chemokine attracting monocytes and macrophages into areas of active infection (Albini et al., 1998b; Rao et al., 2013). Furthermore, most cells analyzed including monocytes, macrophages, microglia, CD4^+^ T lymphocytes, astrocytes, neurons, and cardiomyocytes have been demonstrated to be able to take up Tat protein (Liu et al., 2000; Tyagi et al., 2001; Eugenin et al., 2007; Aksenova et al., 2009; Yao et al., 2010). Their ability to take up Tat has been linked to different surface receptors or proteoglycans such as heparan sulfate proteoglycans (HSPGs), chemokine receptors, integrins, and lipoprotein receptor-related protein-1 (LRP-1) to name a few that can interact with the basic region and the RGD motif on Tat protein. These interactions allow Tat to be endocytosed into bystander cells through two major pathways. First, there is the clathrin-mediated endocytosis pathway, which is dependent on the AP-2/clathrin/dynamin-2 pathway (Vendeville et al., 2004). Vesicles that originate from this canonical pathway depend on acidification for the maturation of the endosomes which can be perturbed by either keeping cells at 4 degrees Celsius or adding ammonium chloride to prevent acidification. Several studies have shown that Tat uptake is significantly inhibited when cells are subjected to either condition, further demonstrating that Tat uptake is dependent on this pathway (Richard et al., 2005; Ruiz et al., 2019). The second pathway is the caveolar pathway, which is an alternative endocytosis pathway for Tat uptake as it was demonstrated for the uptake of Tat-GFP in HeLa as well as CHO cells (Ferrari et al., 2003; Fittipaldi et al., 2003). Immediately Tat is taking up by cells. The protein is mostly seen in the Rab-5 positive late endosome with a pH of <6.0. The release of Tat from the lumen of these vesicles remains unknown but it has been suggested that the same Tryptophan residue (W11) plays a critical role in its release from the lumen of the endosome to the cytosol (Vendeville et al., 2004). The uptake and sometimes, binding of Tat to some surface receptors leads to transcellular signaling, and this has been demonstrated in multiple cell types (Vogel et al., 1993; Rao et al., 2008, 2013, 2014). The ability of Tat to mediate this signaling has been shown to promote pro-inflammation beyond the low level of viral replication that is seen in present-day HIV-infected patients. Thus, further research to understand the role of viral soluble proteins such as Tat is urgent to prevent bystander damage.

## TAT Effects on Immune Cells, Neurons, and Endothelial Cells

As indicated above, Tat has been shown to have chemotactic activities in monocyte-derived dendritic cells (MDDCs), monocytes, and microglia (Lafrenie et al., 1996a,b; Mitola et al., 1997; Benelli et al., 1998; McManus et al., 2000a; Eugenin et al., 2005), but the specific membrane receptors or mechanism that participate in this migration are unknown. It is expected that chemokine receptors participates in this observed Tat-induced chemotaxis. It has been proposed that, because Tat is a highly basic protein, the basic motif may allow it to interact non-specifically with anionic molecules. The limited conformational and structural studies of full length or regions of Tat protein (Bayer et al., 1995) further limit our understanding to discover novel and specific ways to perturb Tat-associated effects with potential cellular targets.

In monocytes, microglia, and MDDCs, Tat induced chemotaxis is blocked by neutralizing antibodies to CCL2 (McManus et al., 2000b; Eugenin et al., 2005), suggesting that Tat-induced chemotaxis is through increased expression and secretion of CCL2. The increased CCL2 mediates chemotaxis of these cells, mostly to the site of infection. CCL2 is, to date, the most potent monocyte chemoattractant and is chemotactic for activated T cells (Yla-Herttuala et al., 1991; Koch et al., 1992; Villiger et al., 1992a,b; Brown et al., 1996), microglia, and macrophages (Cross and Woodroofe, 1999). Experiments indicate that CCL2 is an essential chemokine for monocyte transmigration into the brain parenchyma (Fuentes et al., 1995; Gonzalez et al., 2002) and elevated levels of CCL2 have been detected in the CSF and sera of HIV-1 infected individuals with HAND (Conant et al., 1998; Kelder et al., 1998). However, some of these findings on the rapid release of chemokines like CCL2 may involve rapid release from pre-storage. For instance, the treatment of endothelial cells with histamine resulted in the release of CCL2, eotaxin, GROα, and IL-8 that were stored in small vesicles and Weibel-Palade bodies within 15 min post-treatment (Oynebraten et al., 2004). In another study, the treatment of CD8^+^ cells with anti-CD3 plus anti-CD28 antibodies or PMA plus ionomycin resulted in rapid RANTES (CCL5) release (Catalfamo et al., 2004). These results suggest that, apart from the Tat-induced upregulation at the transcriptional level of some cytokines, especially, beta-chemokines, there could be an alternative mechanism of Tat-induced fast release of chemokines that induces transmigration. In addition to this spontaneous release of stored chemokines, as earlier stated, Tat has been shown to induce the expression of some beta-chemokines of which CCL2 is one.

Also, Huang et al. showed that the addition of Tat to leukocytes significantly increased the expression of CCR5 and CXCR4 in a dose-dependent manner, thereby suggesting that Tat secretion could be making these cells susceptible to HIV-infection (Huang et al., 1998). Tat also reprograms immature dendritic cells to express chemo-attractants for activated T cells and macrophages, suggesting a role for Tat in the differentiation of dendritic cells and in the recruitment of activated cells into areas of infection, where HIV replicates and is released (Izmailova et al., 2003). In addition to these Tat effects, some HIV-infected individuals abuse drugs, which results in enhanced activity of Tat on cell migration, inflammation, and neuronal toxicity (Gurwell et al., 2001; Nath et al., 2002; El-Hage et al., 2005). However, the mechanism that mediates these enhanced effects is unknown. Therefore, further experimental approaches are required to investigate the mechanism behind how drug abuse enhances Tat activities or effects on the multiple HIV associated co-morbidities.

In uninfected cells, the effect of Tat has been explored and shown to be multifaceted. Tat has been shown to affect the large protein quality control machinery, which is one of the protein degradation complexes referred to as the proteasome. Two studies have shown that Tat protein alters the activity of the proteasomal complex to induce increased or decreased ubiquitination and targeting some cellular proteins for degradation while reducing degradation of other cellular and viral proteins. These studies also suggested that there is a direct interaction between Tat and the proteasomal complex which can lead to inhibition of proteasomal activity (Huang et al., 2002; Apcher et al., 2003). These experiments ultimately show that Tat can directly alter the degradation of cellular proteins and enhance the accumulation of viral and perhaps, other cellular proteins that favors the formation of new virions in HIV-infected cells.

**Figure 2:**
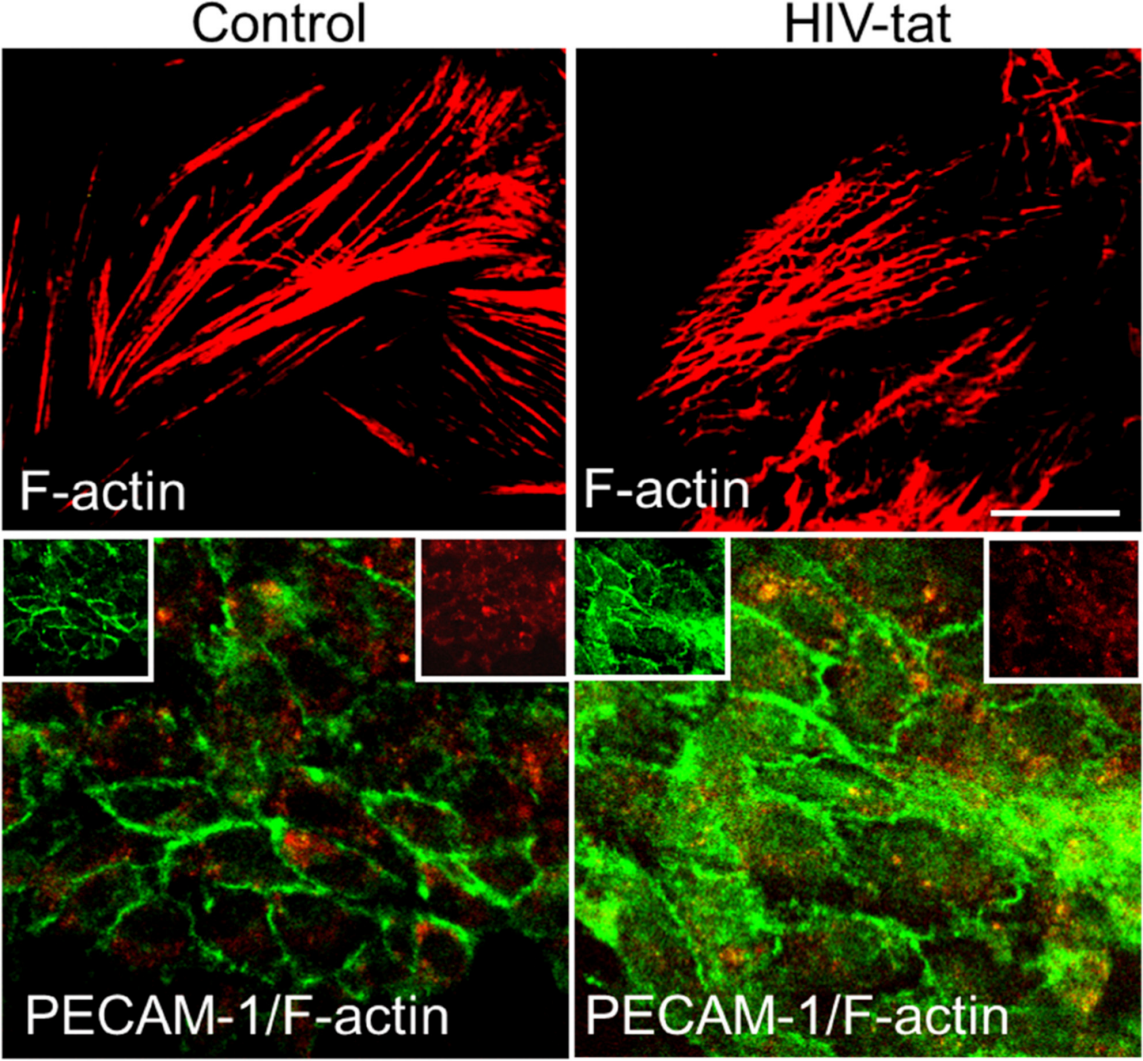
Tat alters actin cytoskeleton and PECAM expression. Cells were treated as empty media and HIV-1 Tat containing media. In control cells, actin cytoskeleton (Cy3-red) remains intact, and PECAM expression (green) is as expected mainly localized to the plasma membrane. In Tat treated samples, actin cytoskeleton is disrupted, and PECAM expression and distribution changes to be in the cytoplasm and at the plasma membrane.

Tat has also been shown to have effects on endothelial cells (ECs), leading to multiple defects through activation of VEGF/KDR receptors, chemokine receptors, HSPGs, and integrins. Recently, it was demonstrated that Tat binds to α_v_β_3_ integrins inducing FAK phosphorylation that triggers the activation of different intracellular messengers (Urbinati et al., 2005). This FAK activation has been associated with migration, enhanced permeability (Avraham et al., 2004), and may explain the alterations in tight junction proteins such as Connexins, Claudin-5, and ZO-1 observed after Tat treatment (Andras et al., 2005; Pu et al., 2005). Tat treatment of endothelial cells can alter the formation of actin filaments, tight junctions, and adhesion molecules, such as ZO-1, JAM-A, PECAM-1, and CD99 (Williams et al., 2013). Results obtained by confocal microscopy demonstrated that, Tat treatment for 12 h induced an aberrant, non-linear, actin filaments, and PECAM-1 is redistributed into the surface of the endothelial cells compared to the untreated cells (Figure 2). These changes, in addition to ECs migration, also can be associated with the pro-invasive activities of Tat. Experiments in transgenic mice or metastatic cells expressing Tat showed an enhanced release of Matrix Metalloproteinases (MMPs) that are associated with leukocytes, monocytes, or metastatic cells infiltration, suggesting that Tat in concert with other factors, enhance cell migration (Albini et al., 1994; Zocchi et al., 1997; Prakash et al., 2000). Another almost unexplored form of migration is the neuronal or stem cell migration. This form of migration is important during the regeneration of tissue and synaptic plasticity. They are both critical in NeuroHIV and are perturbed by Tat protein. First, Tat has been described to: alter nerve growth factor (NGF) signaling pathway, decrease expression of p35, a neuron-specific activator of CDK5, a cyclin that phosphorylates several neuronal proteins involved in cell survival, migration and differentiation (Peruzzi et al., 2002). Specifically, p35, CDK5, and other cyclins have been described to be essential in normal neuronal migration because mice deficient in cdk5 expression have been shown to have perturbed neuronal migration pattern (Ohshima et al., 1996; Gilmore et al., 1998; Gilmore and Herrup, 2001). Also, *in vivo* experiments have shown alterations in FGF-1 and BDNF expression in neurons that survived in the brain of patients that suffered HIV-encephalitis (HIVE) (Soontornniyomkij et al., 1998; Everall et al., 2001). Embarking on studies to understand the potential contribution of decreased neuronal differentiation or migration to the areas compromised with HIV will be an excellent addition to our knowledge on how HIV causes neuro-cognitive decline.

## Mechanism of Neurotoxicity

HIV enters the CNS soon after infection via transmigration of HIV-infected leukocytes and monocytes into the brain (Joseph et al., 2015). Even with cART (Kranick and Nath, 2012), HIV-1 remains in the brain. Patients on cART with no detectable viral load and high CD4^+^ T cell counts still suffer from HAND. It is currently estimated that about 50% of HIV-1 infected individuals have HAND irrespective of their cART status (Rojas-Celis et al., 2019), suggesting a mechanism CNS specific (Kusdra et al., 2002). These HIV-infected cells release Tat that can induce neurotoxicity by two mechanisms; act directly on neurons or act through other cell types like macrophages/microglia and astrocytes, triggering inflammation. The findings that Tat-subtype B, which is the predominant subtype in the USA, but not subtype C (predominant in India), results in significantly higher levels of HIV dementia, 15–30% in the USA compared to 1–2% in India both in pre-therapy era, suggest that neurotoxic potential of Tat protein from different subtypes may vary (Antinori et al., 2007; Heaton et al., 2010, 2011). This finding has been explored by multiple groups, and there have been several studies that have validated this suggestion. Neurotoxic potentials of the Tat protein have been shown by some studies to be associated with some specific molecular signatures that can influence different activities of Tat protein. For instance, Tat plays a key role in the recruitment of monocytes/macrophage cells into the brain and has direct association with HAND (Abraham et al., 2003; Li et al., 2008; Constantino et al., 2011; Bagashev and Sawaya, 2013; Burdo et al., 2013; Chompre et al., 2013; Fields et al., 2015a,b; Niu et al., 2015). It has been shown that Tat with the critical CC motif; mimics chemokines, binds to chemokine receptors, and can recruit monocytes and macrophages to the CNS while Tat without the intact CC motif does not bind to chemokine receptors and it is unable to recruit these cells (Ranga et al., 2004; Mishra et al., 2008). Another molecular signature that has been identified and linked to Tat neurotoxicity is the R57 residue in the basic domain of Tat (Eguchi et al., 2001; Hashida et al., 2004; Ruiz et al., 2019). The basic domain, which is in the same as the cell-penetrating peptide (CPP), mediates cellular uptake of Tat, and the loss of one basic amino acid has been shown by several groups to perturb cellular uptake. A recent study has shown, there is a natural polymorphism seen in Tat mostly in HIV-1 subtype C which significantly reduces cellular uptake of Tat with S57. It was equally shown that less cellular uptake of Tat-S57, translated to less induction of pro-inflammation and less neurotoxicity (Ruiz et al., 2019). On the other side, some studies suggest that Tat protein from subtype C is a better transactivator when compared to subtype B (Kurosu et al., 2002; Johri et al., 2015). These findings show that polymorphisms in Tat can have significant effects on the neurotoxic potential as well as transactivating activity of the protein.

**Figure 3:**
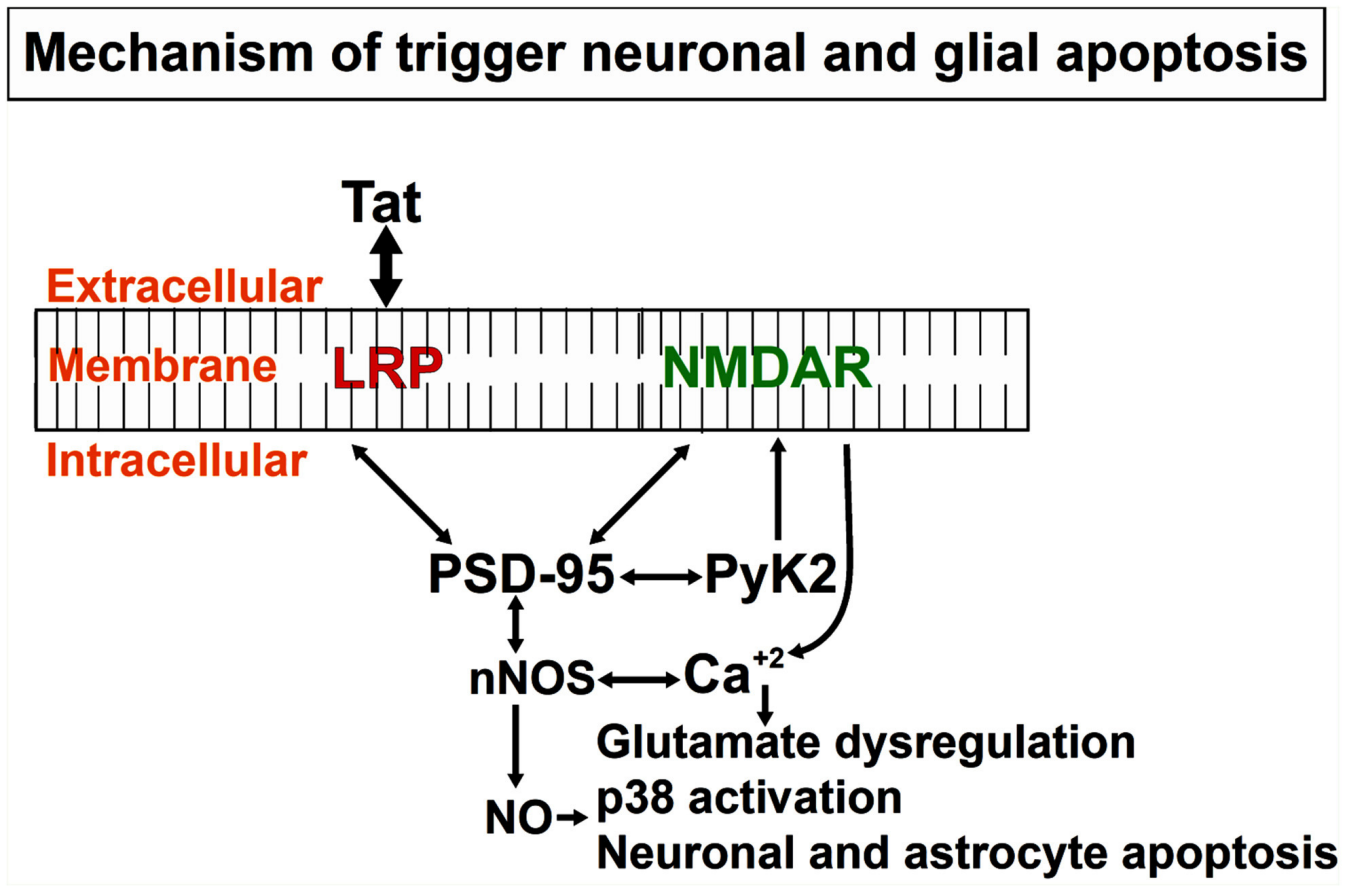
Mechanistic depiction of Tat induced neuronal and astrocytic apoptosis: Tat protein taken up by bystander cells induces the formation of nNOS that leads to the formation of Nitric Oxide (NO), significant release of calcium from intracellular calcium stores that triggers other downstream mediators of apoptosis in both neurons and astrocytes. Apoptosis of both cell types (neurons and astrocytes), as well as others like axonal/dendritic shrinkage, causes a neurocognitive decline in HIV-infected individuals.

**Figure 4:**
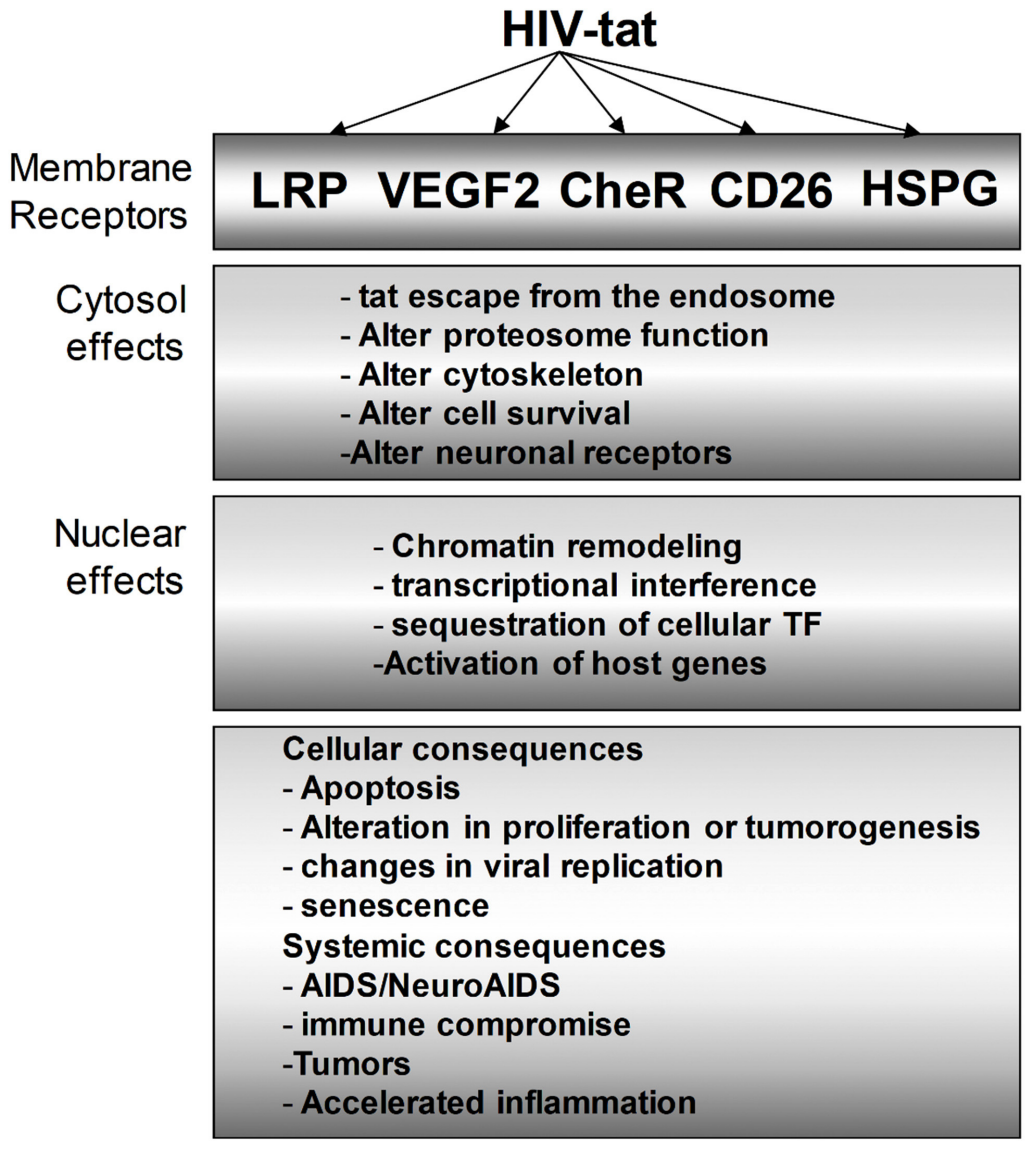
Multiple intracellular mediators of the Tat effect on bystander cells. Tat protein modulates HIV-infected cells as we as uninfected bystander cells. In the cytosol, the effect of Tat ranges from altering the proteasomal degradation pathway to favor viral and host proteins that support viral replication, cell survival, and other identified phenotypes. Similarly, Tat mediates multiple changes in the nucleus to effect transcriptional changes that can vary cellular effects such as apoptosis, inflammation, and many more.

We and others have described the neurotoxic effects of Tat, in part by activation of diverse neuronal players, such as receptors and/or channels like: the N-methyl-D-aspartate (NMDA), non-NMDA receptors, sodium channels, and intracellular pathways (caspase-3) (Eugenin et al., 2007; Li et al., 2008; Aksenova et al., 2009; Hu, 2016). However, conflicting results have been reported, in part due to differences in the culture system, species analyzed, section of the brain examined, and methods to determine the neurotoxicity (Chiozzini and Toschi, 2016). Published data from our laboratory, obtained in human primary neurons indicates that Tat-induced apoptosis is a process that is dependent on glutamate receptor, NMDA receptor, the low-density lipoprotein receptor-related protein-1 (LRP), and nNOS (Eugenin et al., 2003). The intracellular protein that organizes these players is PSD-95. As shown in Figure 3, PSD-95 can interact with different membrane and intracellular proteins, such as LRP, NMDA receptors, nNOS, adhesion molecules, guanylate kinase, and diverse tyrosine kinases, including Fyn, and Pyk2. A critical example of the importance of these interactions in pathological conditions and the potential crossroad with Alzheimer's and Parkinson's diseases is the strong correlation of ApoE4 alleles with dementia and survival in the three different diseases (HAND, Alzheimer, and Parkinson). Currently, in the ART era, HAND has some pathological features that are like Alzheimer's and Parkinson's diseases. For example, ApoE4 is a ligand for the lipoprotein receptor-related protein (LRP), and ApoE4 polymorphisms are associated with poor prognoses in numerous neurodegenerative diseases, including Alzheimer's disease, stroke, hemorrhage, trauma, as well as HIV dementia/neuropathology. However, in HIV-neuropathogenesis, the viral protein Tat has been shown to bind to LRP (Liu et al., 2000), the same receptor that binds ApoE, and to induce similar neurotoxicity as ApoE (Eugenin et al., 2003, 2007). Tat and ApoE may have similar or overlapping signaling pathways, and thus Tat needs to be considered as important in the pathogenesis of dementia in HAND. This Tat-mediated neurotoxicity through LRP-1 signaling pathways involve activation of LRP-1, the receptor for both ApoE and Tat on neurons, NMDAR, a synaptic scaffold protein, that leads to the production of glutamate, as well as nitric oxide (NO) (Eugenin et al., 2007) (see Figures 3, 4). Furthermore, Tat through these mechanisms can amplify inflammation and neurotoxicity at long range due to the diffusion of NO into vast areas of the brain. We propose that these amplification methods can partly explain some of the vast synaptic compromise observed in the majority of the 50% of the HIV-infected individuals with HAND despite effective ART.

The neurotoxicity of Tat also involves apoptosis. Tat-induced apoptosis is a bi-phasic process. First, a process dependent on LRP, PSD-95, and NMDA receptors to mediated Tat-internalization and then the second process to trigger and amplify Tat-toxicity from neurons to astrocytes by a mechanism that involves at least glutamate dysregulation, NO generation and diffusion, as well as calcium overload (Figure 3). Typically, primary neuronal culture has 35 % of NMDA positive neurons and when these cultures are treated with Tat, it results in 80% of apoptosis at 24 h in the neurons and 20 % in the astrocytes suggesting that Tat first target NMDA receptor-positive neurons and then spread apoptotic signals to astrocytes. The use of NMDA receptor blockers reduced apoptosis in both cell types (Eugenin et al., 2003).

Some reports have suggested that Tat can interact directly with NMDA receptors on the Zn^+2^ binding site (Prendergast et al., 2002; Self et al., 2004; Chandra et al., 2005). Another study has shown that Tat can interact with NMDA receptors through the intact CC-motif in its basic domain, which leads to increased calcium release and retraction of synapses during excessive firing (Li et al., 2008). However, there are no conclusive experiments that have shown Tat binding directly to NMDA receptors, especially to the Zn^+2^ sensitive sites. Also, it is known that Tat can induce the activation of inositol 1,4,5-triphosphate (IP_3_), which mobilizes intracellular release of calcium from the endoplasmic reticulum (ER) stores (Borgatti et al., 1998; Haughey et al., 1999, 2001; Mayne et al., 2000; Feligioni et al., 2003; Fotheringham et al., 2004). This excessive calcium release contributes to neurotoxicity. Another mechanism of Tat-induced neurotoxicity is the interaction of Tat and LRP-1, thereby causing the internalization of LRP-1 and decreased uptake of LRP-1 ligands, in this case, amyloid-β peptide and Apolipoprotein E. Data from our laboratory and others have shown that specific LRP blockers like receptor-associated protein (RAP) that is an endoplasmic chaperone for LRP-1 and tightly binds the NMDA receptor when applied extracellularly, block the binding, and prevents the uptake of all known LRP ligands (Prasad et al., 2015), including Tat (Herz and Strickland, 2001; Strickland et al., 2017), suggesting, almost all the initial toxic effects of Tat are mediated by LRP, but we cannot discard a cross-activation or sensitization of NMDA receptors as an additional mechanism (see Figure 4).

A report established a connection between the dementias induced by HIV-infection and Alzheimer's with regards to neprilysin (NEP). NEP is the major enzyme that degraded Aβ in the brain. NEP is a membrane surface zinc metalloendopeptidase, also known as CD10, that cleave bradykinin, substance P, and insulin. Tat has been shown to inhibit NEP (Turner et al., 2001). This inhibition results in increased Aβ levels and its accumulation. Besides, it was demonstrated that individuals with long term HIV-infection had increased Aβ. Also, LRP has been associated with controlling extracellular activities for MMPs (Emonard et al., 2005). Both LRP and NEP are considered risk factors in Alzheimer's disease and may equally have connections with the mechanism of HAND.

We think that Tat enhances or induces the formation of apoptosis promoting complex in the surface of neurons made up of Tat, LRP, PSD-95, NMDAR, nNOS, and Pyk2. While there is no Tat in AD, we speculate that a similar complex might be formed in AD and associated with either the beginning or progression to AD. ApoE4 is another factor that induces significant low levels of apoptosis in neurons and astrocytes compared to control cells but is not comparable to the apoptosis induced by Tat. The association of ApoE4 polymorphisms with worse prognoses in numerous neurodegenerative diseases, such as AD, stroke, hemorrhage and HIV dementia, and neuropathy (Strittmatter et al., 1993; Roses et al., 1995; Slooter et al., 1997; Corder et al., 1998), suggest a key role of LRP and their ligands in association with dementia. Intracerebroventricular injection reduces LTP by an NMDA dependent mechanism, suggesting an alteration in neurons. Tat has also been shown to have effects on lipid peroxidation, which are significantly reduced while the generation of reactive oxygen species (ROS) is increased in neurons and synaptosomes from APOE4 KO mouse, suggesting there could be a mechanism that modulates the levels of these two factors. A study demonstrated that Tat and APP colocalize in the brains of SIV-infected macaques suggesting again that may these two diseases share toxic pathways to generate dementia.

However, why is Tat different from other LRP ligands that trigger massive apoptosis? One of the key differences that can be observed from the calcium imaging recording experiment shows, Tat induces general dysregulation of intracellular calcium (Haughey et al., 1999) compared to ApoE4 treatment, but not α_2_M or lactoferrin, which results in modest neurotoxicity (Qiu et al., 2003). Also, in contrast to other LRP ligands, Tat works differently, because it induces the recruitment of the membrane protein-like NMDA receptor, PSD-95, nNOS, and Pyk2 to form a membrane complex that amplifies apoptosis, not observed with other LRP ligands (Figure 3). Another difference is that, Tat after it internalization can escape from endosomes and localize to the nucleus where it can alter transcription of cellular genes suggesting that Tat, after internalization can alter the synthesis of key proteins such as transcription of synaptic (Eugenin et al., 2003) or survival proteins that finally trigger massive apoptosis. However, we think one of the key processes of Tat-induced neurotoxicity is the interaction of Tat with NMDA, glutamatergic neurons because early blocking of these channels or blocking the formation of the complex (especially nNOS activation) results in a reduction of Tat toxicity. All these possibilities are under current investigation in our laboratory. We speculate that under normal conditions, this complex exists, and the addition of or exposure to Tat only enhances the complex by increasing the recruitment of more factors involved in the formation of this complex at the membrane.

Published studies from our laboratory show that Tat treatment leads to an elevation in intracellular calcium of human fetal neurons and we observed that this Tat-induced calcium elevation is biphasic in both neurons and astrocytes (King et al., 2006). Also, this calcium elevation is characterized as a single, sustained, and oscillatory spike. We think the cascade of activities that leads to the calcium elevation is; first calcium from IP_3_-dependent release and a subsequent sustained glutamate receptor-mediated mechanism (Haughey et al., 1999, 2001). But it is still unclear in human cells, if glutamate activity can alter IP_3_-activity like the report in chicken neurons which demonstrated that glutamate could modulate IP_3_-mediated calcium release. In the same direction, other published works from our laboratory have shown that early NMDA glutamate receptor activation is not enough to induce neuronal apoptosis because the addition of MK801 after 3 h of Tat treatment still blocks apoptosis, suggesting that early calcium spike is not related to apoptosis. So far, no study has established or demonstrated the link between IP_3_, NMDA receptor, astrocytes, and apoptosis, as well as gap junctions. However, gap junctions are permeable to IP_3_ and calcium. Also, gap junctions control glutamate metabolism and are a critical communication system between neurons-neurons and neurons-astrocytes (Eugenin et al., 2007). We speculate that the co-application of Tat and gap junction blockers will reduce Tat-induced apoptosis. This will suggest that gap junctions are actively involved in transmitting secondary messenger(s) that may mediate bystander killing to other cells (that are not exposed to Tat but receive these secondary messengers. While we do not know this secondary messenger, glutamate could be a good candidate because it is known to mediate neurotoxicity. Identifying this secondary messenger will be a significant finding that may potentially open the avenue for tackling HAND.

Tat has also been shown to affect the expression of some miRNAs in neurons. For instance, Tat upregulates the expression of miR-34a, a miRNA that targets CREB (Zhang et al., 2012; Zhan et al., 2016). The upregulation of miR-34a leads to the promotion of neuronal dysfunction (Hu et al., 2017; Periyasamy et al., 2019). It also has been proposed that Tat can mediate indirect neuronal toxicity by inducing microglia/macrophagic and/or astrocytes to release toxic factors such as cytokines, chemokines and others (Eugenin et al., 2005; El-Hage et al., 2006a,b; Bagashev and Sawaya, 2013; Joseph et al., 2015; Hu et al., 2017; Periyasamy et al., 2019). The indirect neurotoxic effect of Tat is based on its ability to be taken up by uninfected bystander cells. One's Tat protein is endocytosed, it is released from the endosome as the endosome undergoes maturation. After the release of Tat from the lumen of endosomes to the cytosol, Tat interacts with multiple intracellular factors, which lead to modulation and changes in cellular response. Many of the interacting cellular factors have been extensively discussed in several studies and reviews. Tat is also known to cause transcriptional changes that lead to either downregulation or up-regulation of target genes.

For example, Tat interacts with the NF-κB inhibitor IκB-α, that leads to the release of NF-κB and its translocation into the nucleus. Translocation of NF-κB leads to the transcription of several genes (Fittipaldi and Giacca, 2005; Easley et al., 2010; Zhang et al., 2012). Some of these target genes have significant effects on HIV-1 pathogenesis. Specifically, Tat has been shown to upregulate transcription of genes like CXCR4, CCR5 in PBMCs, which are critical for spreading HIV-infection (Huang et al., 1998; Zheng et al., 2005). Also, some pro-inflammatory cytokine genes like TNF-α, CCL2, and anti-inflammatory genes like IL-4 and IL-10 in MPs. Tat has also been linked with inducing anti-proliferation factors from macrophages, which can suppress the activation of naïve CD4^+^ T cells and downregulate anti-apoptotic proteins. This ability of Tat to induce both pro- and anti-inflammatory factors is one of the reasons for some controversies associated with the role of Tat in HIV- pathogenesis (Gandhi et al., 2009).

However, there are no controversies in the role of Tat in HAND. The direct and indirect neurotoxic effect of Tat has been well-established, as seen in the significant reduction in cell viability. In the CNS, the indirect effect of Tat has been characterized by several studies that revealed secreted Tat from HIV-infected cells are taken up by uninfected bystander cells like microglia and astrocytes. For instance, in microglia and astrocytes, uptake of Tat leads to the upregulation and/or release of several neurotoxic factors. The released neurotoxic factors damage the neuron in addition to the direct damages. The secretion of neurotoxic factors mostly in the form of cytokines like TNF-α, IL-6, IL-8, IL-1β, and CXCL1, among others (Ruiz et al., 2019). That is not to say, cytokines are the only neurotoxic factors that mediate Tat indirect neurotoxic effects.

Only recently, we identify that a key mechanism of bystander apoptosis and a neuronal and endothelial compromise was mediated by gap junction channels. Usually, gap junction channels are shutdown under inflammatory conditions (Eugenin et al., 2007; Berman et al., 2016). We found that latently HIV-infected astrocytes maintain connexin43 expression despite the inflammatory phenotype (Berman et al., 2016). Connexin43 (Cx43) formed functional Cx43 containing gap junction channels that enable small molecules generated in latently HIV-infected astrocytes to diffuse into neighboring uninfected cells. We identify that some of these toxic molecules corresponded to calcium, IP_3_, and cytochrome C related signals; however, the mechanism of dysregulation and diffusion of these molecules is unknown. To identify the viral component(s) involved in the Cx43 maintenance, we tested all HIV proteins in uninfected cultures of human astrocytes. Only Tat increased expression of Cx43 and increased cell-to-cell communication enabling the toxic signals generated in the few latently infected astrocytes to diffuse longer distances as compared to uninfected cultures. Also, we observed that Tat binds to the Cx43 promoter and keep its mRNA expression high. This finding is unique and explains the large areas compromised by HIV reservoirs even in the current ART era. In conclusion, the combination of direct and indirect effects of Tat leads to neurobehavioral deficits, which are one of the hallmarks of HAND (Prevedel et al., 2017).

## Mechanism of Tat-Mediated Cardiovascular Disease

Since HIV-infection is now a chronic disease, several comorbidities like atherosclerosis and cardiovascular disease all related to aging have become common within the population of people with HIV (Gebo et al., 2010; Clifford and Ances, 2013; Fields et al., 2013; Buggey et al., 2019; Ciccarelli et al., 2019). It is known that HIV-infected individuals have 1.5–2 times higher risk of cardiovascular disease (CVD) due to several factors like chronic inflammation, dyslipidemia, lipid abnormalities like low high-density lipoproteins (HDL), increased triglycerides, and prolonged use of ART (Wang et al., 2015; Zungsontiporn et al., 2016; Prevedel et al., 2017; Ciccarelli et al., 2019; Estrada et al., 2019; Heravi et al., 2019; Palma Reis, 2019). HIV-associated CVD is characterized by extensive loss of cardiomyocytes, increase in fibrous tissues and significant infiltration of immune cells to the heart (Prevedel et al., 2017). Most of the studies that have examined the effect of HIV-infection on CVD have shown that there is an increased rate of coronary events and another cardiomyopathy in HIV-infected individuals (Thompson-Paul et al., 2019). The only debate that is still lingering is, does ART contributes to CVD or not and if it does, to what extent does it contribute. A study of a multicenter AIDS cohort study (MACS) showed that there was a significant reduction in LDL and HDL from HIV-infected individuals. Commencement of ART in these individuals led to a significant increase in total cholesterol and LDL to pre-infection levels while HDL remained low (Heravi et al., 2019). Interestingly, the strategies for management of antiretroviral therapy (SMART) study that examined the effect of continuous HIV-replication on CVD events showed that continuous HIV replication in individuals that discontinued therapy increased the risk of CVD compared to individuals that adhered to their therapy, clearly showing the effect of HIV replication on CVD (Siwamogsatham et al., 2019).

In a recent study, HIV-infected individuals with detectable viral loads and low CD4 T cell counts were found to have an increased risk of heart failure, increased risk of cardiovascular death when compared to HIV-infected patients with high CD4^+^ T cell counts and undetectable viral loads (Siwamogsatham et al., 2019). This confirms that HIV, on its own, is a risk factor for CVD either with or without ART.

Cardiomyocytes are the most abundant cells in the heart with some fibroblasts. These cells are not infected by HIV, but they can be exposed to both the full virus or specific viral proteins that are shed by HIV-infected cells. Similarly, as is the case with NeuroHIV, uninfected bystander cells that are exposed to HIV or shed viral proteins like Tat and gp120 can secrete pro-inflammatory factors which can be toxic to cardiomyocytes and the heart (Coll et al., 2006; Wang et al., 2015; Zungsontiporn et al., 2016; Anand et al., 2018; Jiang et al., 2018). Also, the vascular tissue, which is important for the cardiovascular system, is made up of endothelial cells that are susceptible to cytotoxic factors induced by HIV-infections or viral proteins. Also, infiltration of the heart by macrophages and lymphocytes in response to the presence of HIV or HIV protein can contribute further to the assault of cardiomyocytes (Zungsontiporn et al., 2016). Therefore, the combination of HIV-replication, shed viral proteins, infiltration by infected and uninfected macrophages-lymphocyte, pro-inflammatory, and cytotoxic factors shed by bystander cells as well as the lipid dysregulation combines to significantly cause systemic dysregulation that leads to cardiomyopathies.

Several studies have shown that gp120 and Tat, two shed viral proteins that are seen in patients, can cause activation of some pathways that lead to apoptosis of cardiomyocytes and endothelial cells. Inhibition of these pathways, for instance, the MAPK/ERK kinase pathway (MEK inhibitors), prevents apoptosis of cardiomyocytes, and endothelial cells (Lee et al., 2011; Cipolletta et al., 2015). The role of Tat, specifically in HIV-associated CVD, is becoming more appreciated as multiple studies have shown that Tat causes transcriptional changes in different cell types that make up the heart, and some of these transcriptional changes are similar to what has been observed in some non-HIV-associated CVDs. Cardiomyocytes, as the predominant cell type of the heart is the cell type that contracts and relaxes in response to action potentials that must be synchronized effectively from the first cell to the last. The speed of contraction and relaxation requires more effective cell-to-cell communication than diffusion. Therefore, cardiomyocytes have robust cell-to-cell connections in the form of gap junctions that allows for the efficient spread of signals from the initial cardiomyocyte to the last. These gap junctions are at the intercalated discs in between cardiomyocytes, and they are made up of several proteins that allow the formation of these junctions (Zhao et al., 2019). While some of these proteins are anchoring proteins at the plasma membranes, others make intercellular contacts with neighboring cells. One protein family known to be involved in intercellular contacts is the connexins and Cx43, particularly localized mainly at the intercalated discs (Prevedel et al., 2017; Macquart et al., 2018; Schultz et al., 2019). We have shown in a recent publication that Tat induces the upregulation of Cx43 mRNA and proteins in cardiomyocytes and increases in levels of lipofuscin, a known aging heart biomarker, and both observations are seen in non-HIV CVDs as well (Prevedel et al., 2017).

Interestingly, in human hearts, it was also observed that there was lateralization of the increased Cx43 protein expressed in response to Tat exposure, suggesting that Cx43 that under normal condition is localized at the intercalated discs is either targeted to another part of cardiomyocytes or the machinery that targets Cx43 to the intercalated discs has been perturbed. The consequence of mis-localizing Cx43 away from the intercalated discs will be, impairment of cell to cell communication, which is critical for synchronization of contraction and relaxation of the heart. Furthermore, Tat-induced apoptosis of cardiomyocytes led to the formation of plaques of fibrous tissues, accumulation of mitochondria, which suggest the cardiomyocyte apoptosis is through a mitochondria-controlled pathway (see Figure 4).

We and others identified a significant increase in the levels of secreted ATP by a hemichannel dependent mechanism (Seror et al., 2011). High circulating levels of ATP correlates with cognitive impairment and endothelial damage (Seror et al., 2011). Furthermore, the exposure of cardiomyocytes to increasing concentration of ATP *in vitro* caused a significant increase in action potentials, shortening of the time between each contraction and calcium flux in the cells. These findings show that HIV-infection and viral protein exposure lead to multiple damages to the heart and CVD (Prevedel et al., 2017; Tahrir et al., 2018).

The endothelium, made up of endothelial cells, is another part of the cardiovascular system that is known to not support active replication of HIV but, it is exposed to both viral proteins and several inflammatory factors released by both infected cells and bystander cells. The effect of such exposure ranges from increased adhesiveness, permeability, apoptosis, oxidative stress, and more cytokine secretion which can lead to recruitment of lymphocytes, monocytes, and macrophages and the initiation of atherosclerosis (Wang et al., 2015; Anand et al., 2018; Chen and Dugas, 2019). Our group determines that HIV can infect cells of the vascular wall such as smooth muscle cells that contribute to the accelerated atherosclerosis observed in the HIV-infected population (Eugenin et al., 2008). In these samples obtained from HIV-infected individuals, a concentric proliferation of smooth muscle cells was observed, a unique characteristic of the plaque that is not observed in uninfected individuals. Thus, we propose that this mechanism of accelerated atherosclerosis also contributes to heart dysfunction in the HIV-infected population.

## Conclusion and Future Perspectives

This review points to the multiple roles of Tat in HIV-replication and HIV-associated comorbidities with specific emphasis on replication, NeuroHIV, and HIV-associated CVD. Various studies discussed in this review have shown that secreted Tat contributes to all if not most of these comorbidities through complex intracellular interactions that lead to chronic immune activation and inflammation that assaults both the CNS and the cardiovascular system. The HIV-infected population is aging faster than their uninfected age-matched pairs due to the assault of all systems by HIV and viral proteins like Tat in multiple ways as shown in Figure 4. More work is needed in understanding how HIV and viral proteins, especially Tat, affects all systems particularly, CNS and cardiovascular system. With increasing longevity of HIV-infected individuals, there is a need to find therapies that will allow them to live normal or near-normal lives with little to no HIV-associated pathologies. The need to identify useful biomarkers for quick diagnosis and drugs for better therapy cannot be overstated to treat and reverse this population that is aging faster than age-matched pairs.

## Author Contributions

All authors listed have made a substantial, direct and intellectual contribution to the work, and approved it for publication.

### Conflict of Interest

The authors declare that the research was conducted in the absence of any commercial or financial relationships that could be construed as a potential conflict of interest.
